# Granulocytic Sarcoma Presenting as a Palpable Breast Lump

**DOI:** 10.3389/fsurg.2016.00067

**Published:** 2017-01-23

**Authors:** Victor Fernandes Vieira, Quoc Duy Vo, Jean Bouquet de la Jolinière, Fathi Khomsi, Anis Feki, Henri-Marcel Hoogewoud

**Affiliations:** ^1^Department of Radiology, HFR Fribourg – Hôpital Cantonal, Fribourg, Switzerland; ^2^Department of Gynecology, HFR Fribourg – Hôpital Cantonal, Fribourg, Switzerland

**Keywords:** acute myeloid leukemia, myeloid sarcoma, granulocytic sarcoma, chloroma, breast cancer

## Abstract

We report the case of a 45-year-old woman who palpated a voluminous painless lump in the superior outer quadrant of her left breast. Her past medical history revealed an acute myeloid leukemia (AML) treated and considered in remission 1 month prior to this discovery. Imaging work-up by mammogram, US, and MRI showed multiples masses suspect of malignancy in both breasts. US-guided needle biopsy was performed in the palpable mass and in one of the multiple lesions located in the right breast. Histologic findings were compatible with a granulocytic sarcoma in both breasts, which was considered as a relapse of the AML treated a few months earlier.

## Introduction

Granulocytic sarcoma (GS), also known as myeloid sarcoma or chloroma, is a rare extramedullary manifestation of myeloid hematological malignancies mostly associated with acute myeloid leukemia (AML). It is characterized by the formation of clinically evident tumors containing immature myeloid cells in extramedullary sites, which commonly include the skin, soft tissues, the CNS, and the urogenital tract. Although the majority of cases arise in patients with AML, the disease may rarely manifest without or prior to medullary involvement (1–6).

Breast GS is very rare and only accounts for approximately 8% of cases (2). Clinical findings are non-specific and diagnosis can be very challenging, especially in cases presenting with primary breast involvement and no evidence of medullary disease (7, 8).

Despite of its importance for the initial assessment of the disease, breast imaging also lacks specificity and GS can mimic several other tumors, including breast carcinoma and lymphoma. Moreover, only a few reports focusing on radiographic description have been published so far (9–13). Currently, only histological examination associated with immunohistochemistry is capable of confirming the disease (6, 8, 14).

Here, we report the case of a patient presenting with bilateral multicentric breast GS after a few months of confirmed AML remission with special focus on mammographic, sonographic, and MRI findings.

## Case Report

A 45-year-old woman noticed a sore palpable mass located in the outer upper quadrant of her left breast. Clinical examination revealed a painful and hard palpable mass measuring about 2.5 cm × 2 cm with no associated lymph nodes in the left axilla. Her medical history revealed an AML diagnosed 2 years earlier and classified as M4/M5 according to the French–American–British classification with FLT3-ITD and NPM1 mutations. No AML1-ETO, CBFb-MYH11, or PML-RARa aberrations were found. Complete remission was demonstrated by bone marrow examination after induction chemotherapy followed by consolidation chemotherapy with cytarabine and idarubicine.

**Figure 1:**
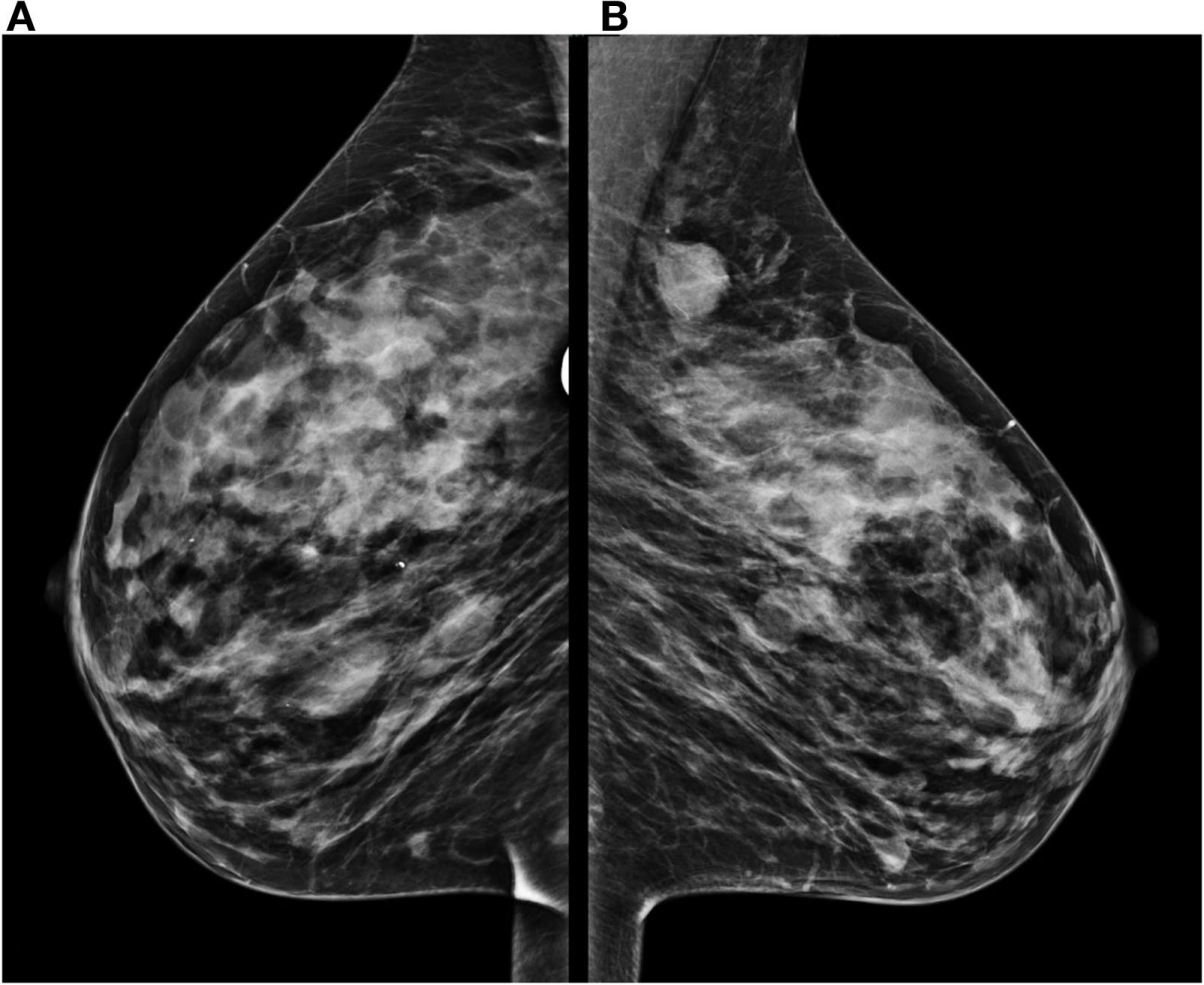
**Mammogram performed with mediolateral oblique incidences of both breasts shows multiple oval well-defined masses located in the right breast (A) and a round lesion with fine spiculated borders located on the superior quadrant of the left breast (B) associated with smaller round lesions**.

**Figure 2:**
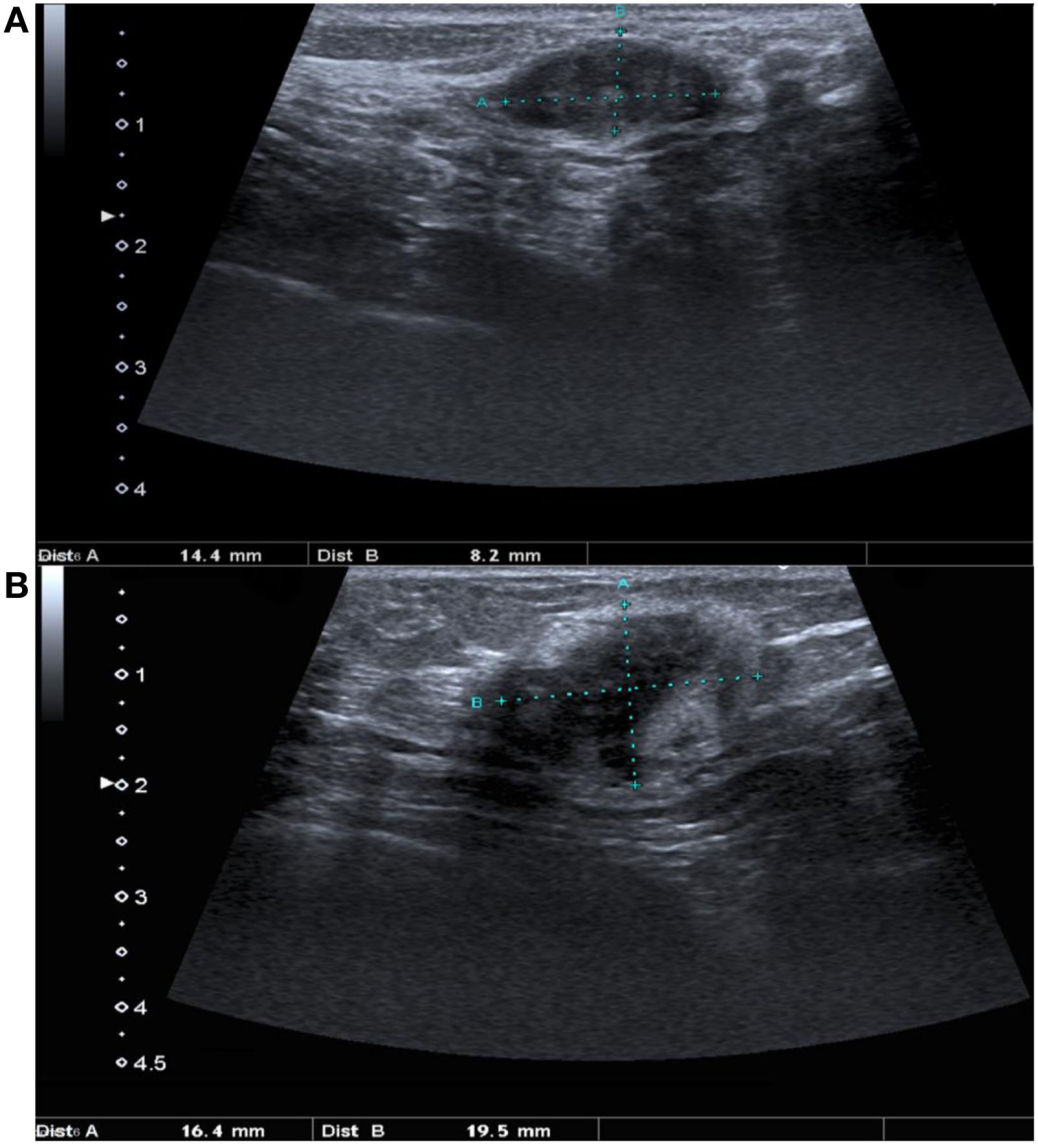
**Ultrasound of both breasts demonstrates one of the multiple masses located on the right breast (A) which is characterized by an oval shape, smooth borders and a hypoechoic appearance**. The palpable mass located in the upper outer quadrant of the left breast **(B)** presents an irregular shape and borders with posterior shadowing suggestive of malignancy.

**Figure 3:**
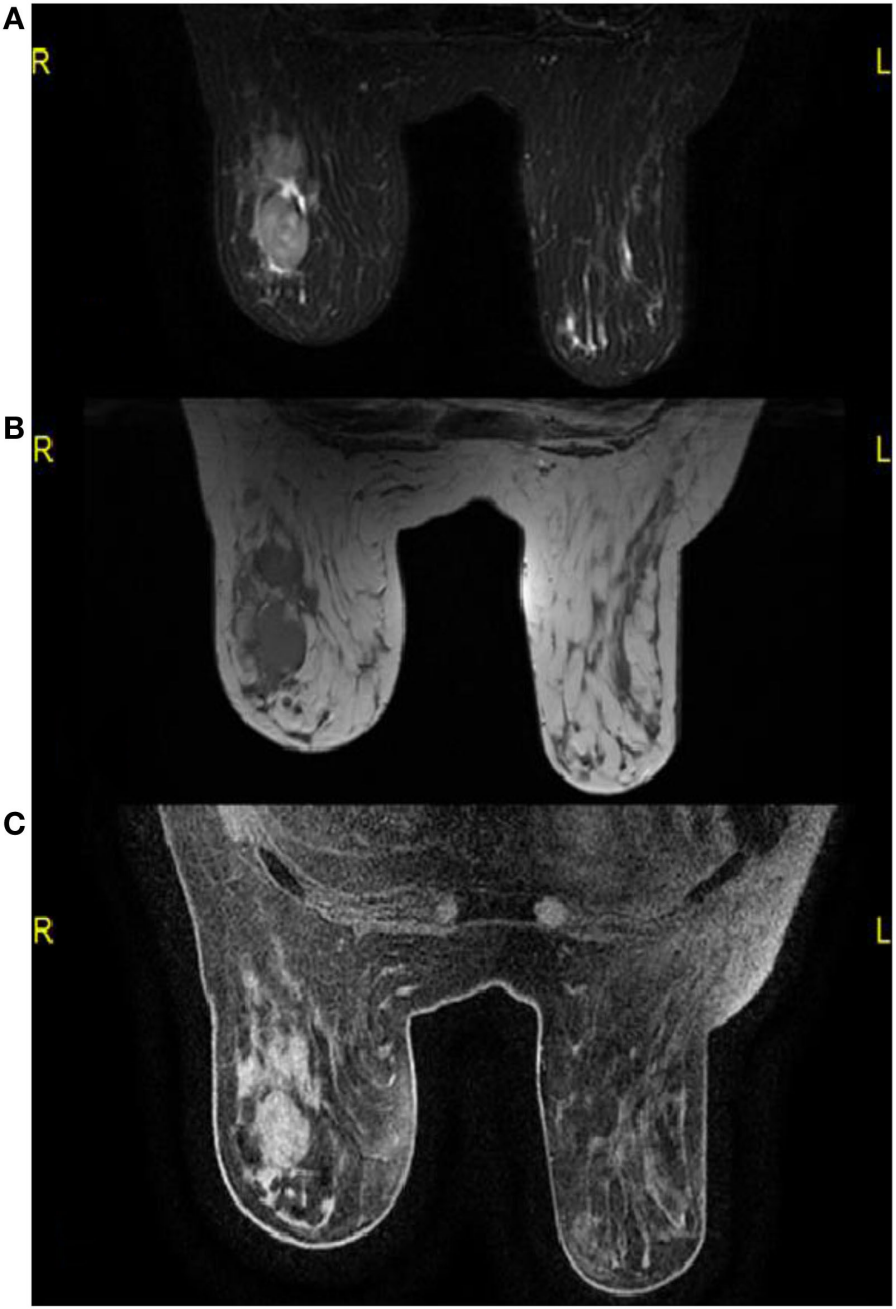
**On MRI, multiple round masses are localized in the outer upper quadrant of the right breast, which present a hyperintense signal on T2-weighted images and fat saturation (A) and a hypointense signal on T1-weighted images (B)**. These lesions present a faint contrast enhancement on gadolinium-enhanced T1-weighted images with fat saturation **(C)**.

**Figure 4:**
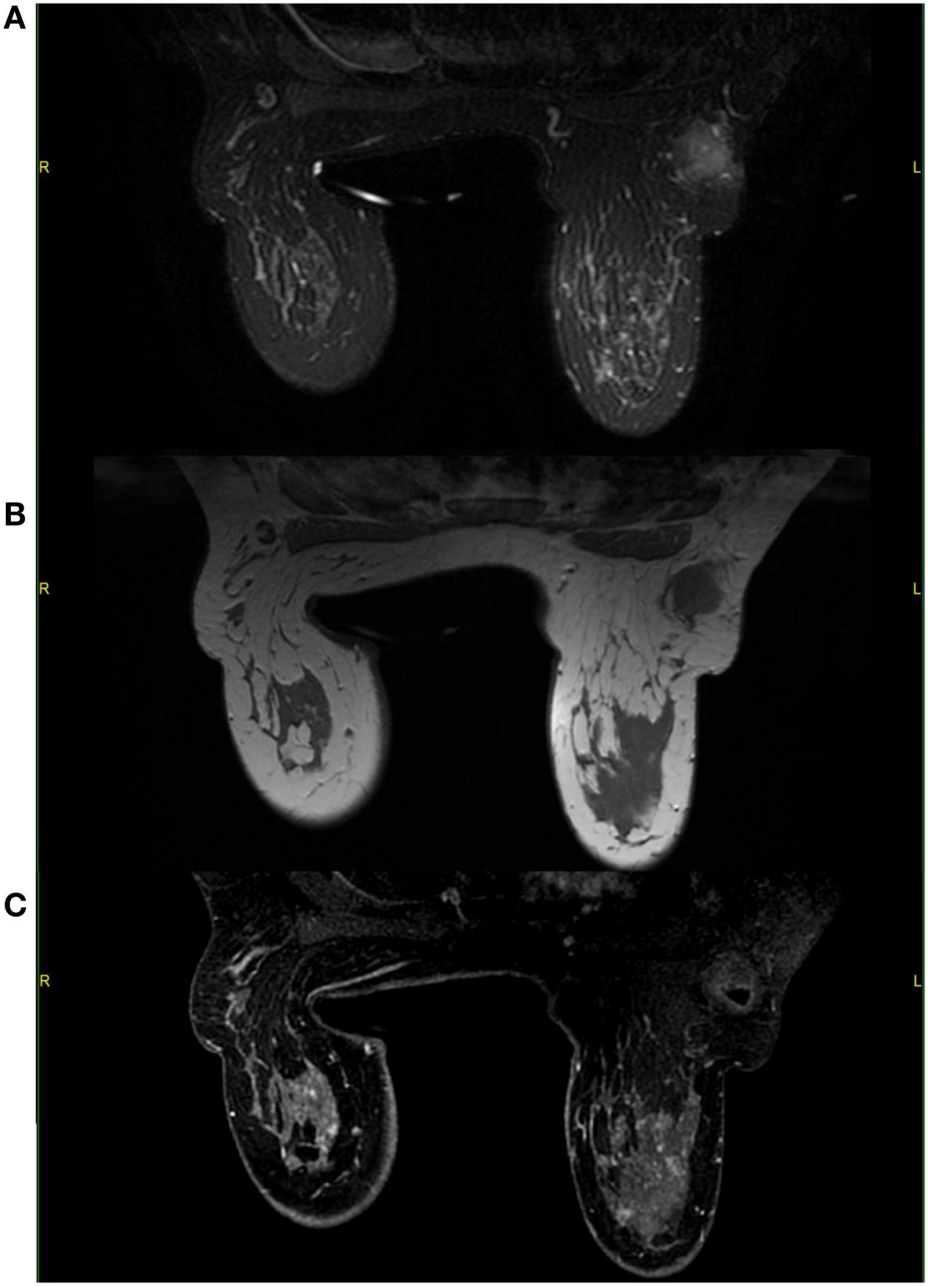
**On MRI, the palpable mass located in the upper outer left quadrant demonstrates a hyperintense signal on T2-weighted images with fat saturation (A) and a hypointense signal on T1-weighted images (B)**. After gadolinium administration, the mass shows little enhancement and a small necrotic area with enhancing borders **(C)**.

A mammogram was performed with only mediolateral oblique incidences because of breast discomfort (Figure 1). It showed a round mass with spiculated margins located in the superior quadrant measuring approximately 2 cm × 1.8 cm and associated with other well-defined oval shaped lesions involving both breasts with a size range of 1–2 cm. An ultrasound revealed an ill-defined hypoechoic lesion with acoustic shadowing located in the outer upper quadrant of the left breast measuring about 1.9 cm × 1.6 cm and corresponding to the known palpable mass. Multiple similar well-defined hypoechoic oval masses were observed scattered throughout the remainder of the breasts tissue (Figure 2). On MRI, all lesions (Figure 3) including the palpable one (Figure 4) were hypointense on T1-weighted images and hyperintense on T2-weighted images. After injection of gadolinium-based contrast medium, most lesions demonstrated a faint enhancement measuring approximately 1–3 cm. On the other hand, the palpable lesion in the left breast demonstrated a “ring” enhancement reminiscent of an abscess. The differential diagnosis of imaging findings include multicentric breast carcinoma, malignant lymphoma, and less likely multiple fibroadenomas.

**Figure 5:**
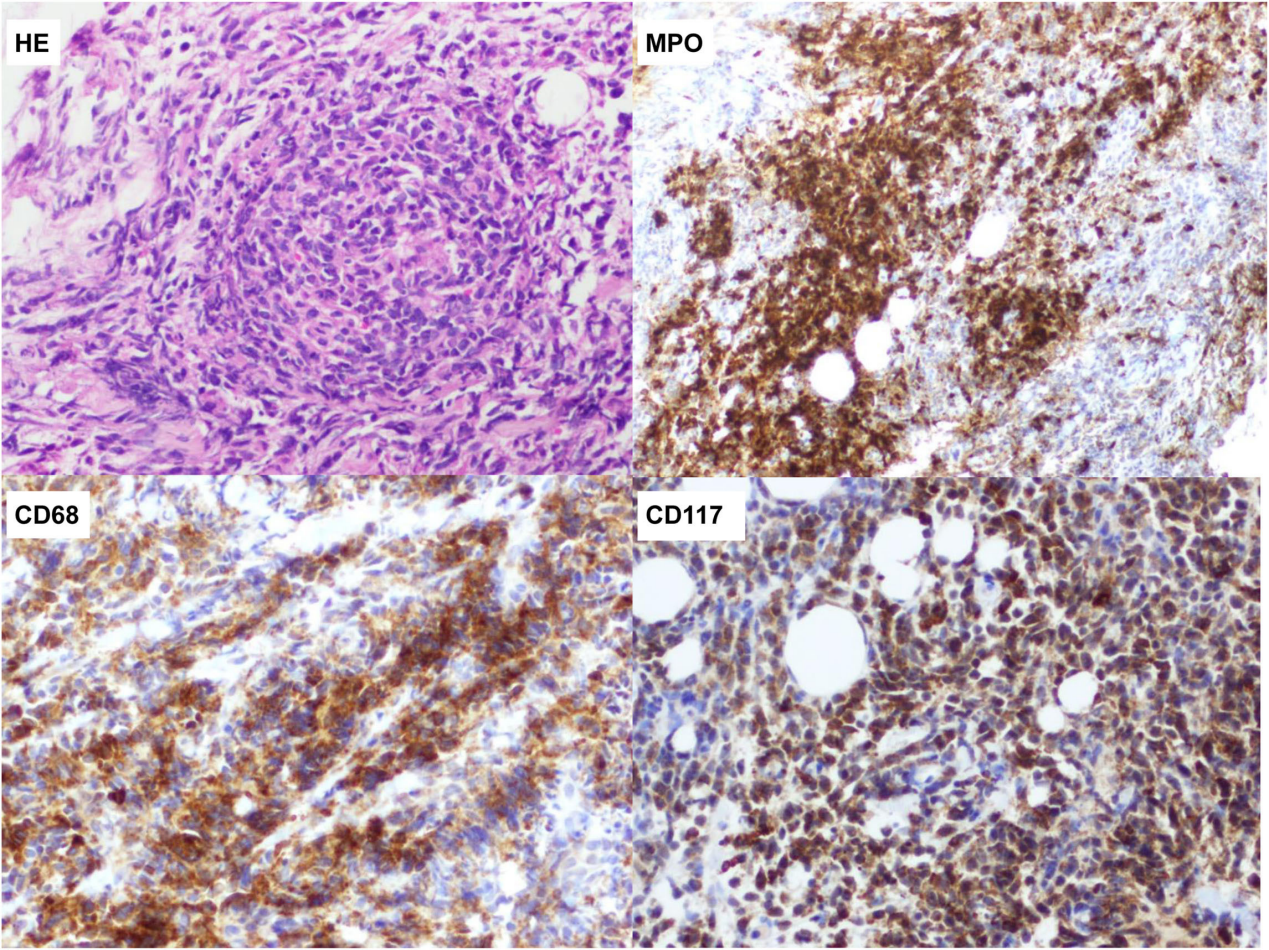
**Dense myeloid precursor proliferation with breast tissue invasion**. Immunostaining is positive for myeloperoxydase, CD68 and CD117, which are markers for myeloid cells.

Lastly, US-guided needle biopsy was performed in the known palpable mass and in one of the multiple lesions located in the right breast. Histological samples showed dense myeloid cellular proliferation with breast tissue invasion. Biopsies were positive for myeloperoxydase (MPO), CD68, and CD117, which are markers for myeloid tumors (Figure 5). Molecular biology analysis resulted positive for FLTD3-ITD mutation. Histological features were consistent with extramedullary AML of both breasts.

**Figure 6:**
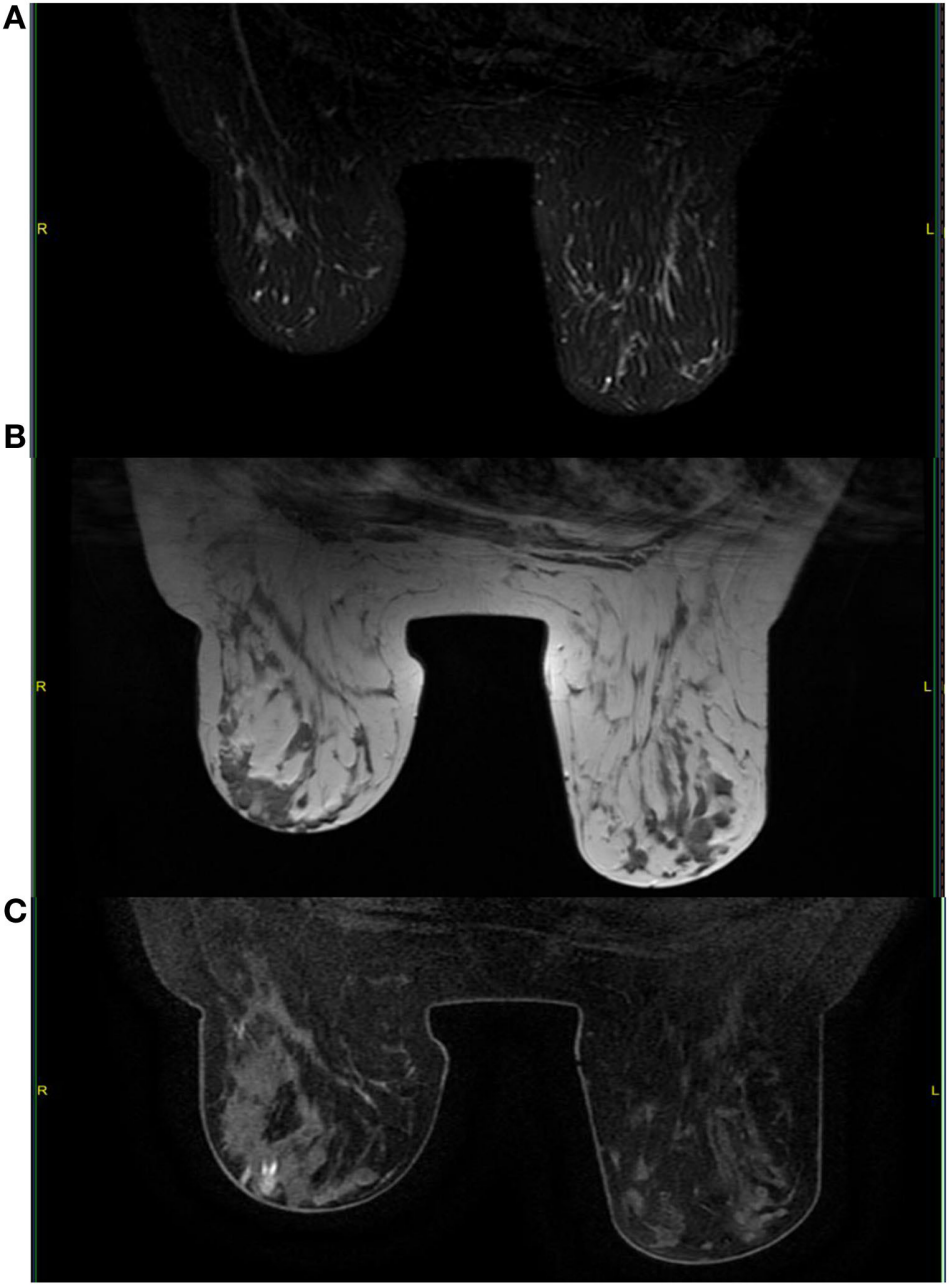
**Follow-up with MRI shows significant decrease in tumor burden with no evidence of new lesions**. A complete response was achieved in most lesions and observed by the absence of contrast enhancement especially in the big cluster of oval masses of the right breast (arrow). T2-weighted images with fat saturation **(A)**; T1-weighted images **(B)**; gadolinium-enhanced T1-weighted images with fat saturation **(C)**.

**Figure 7:**
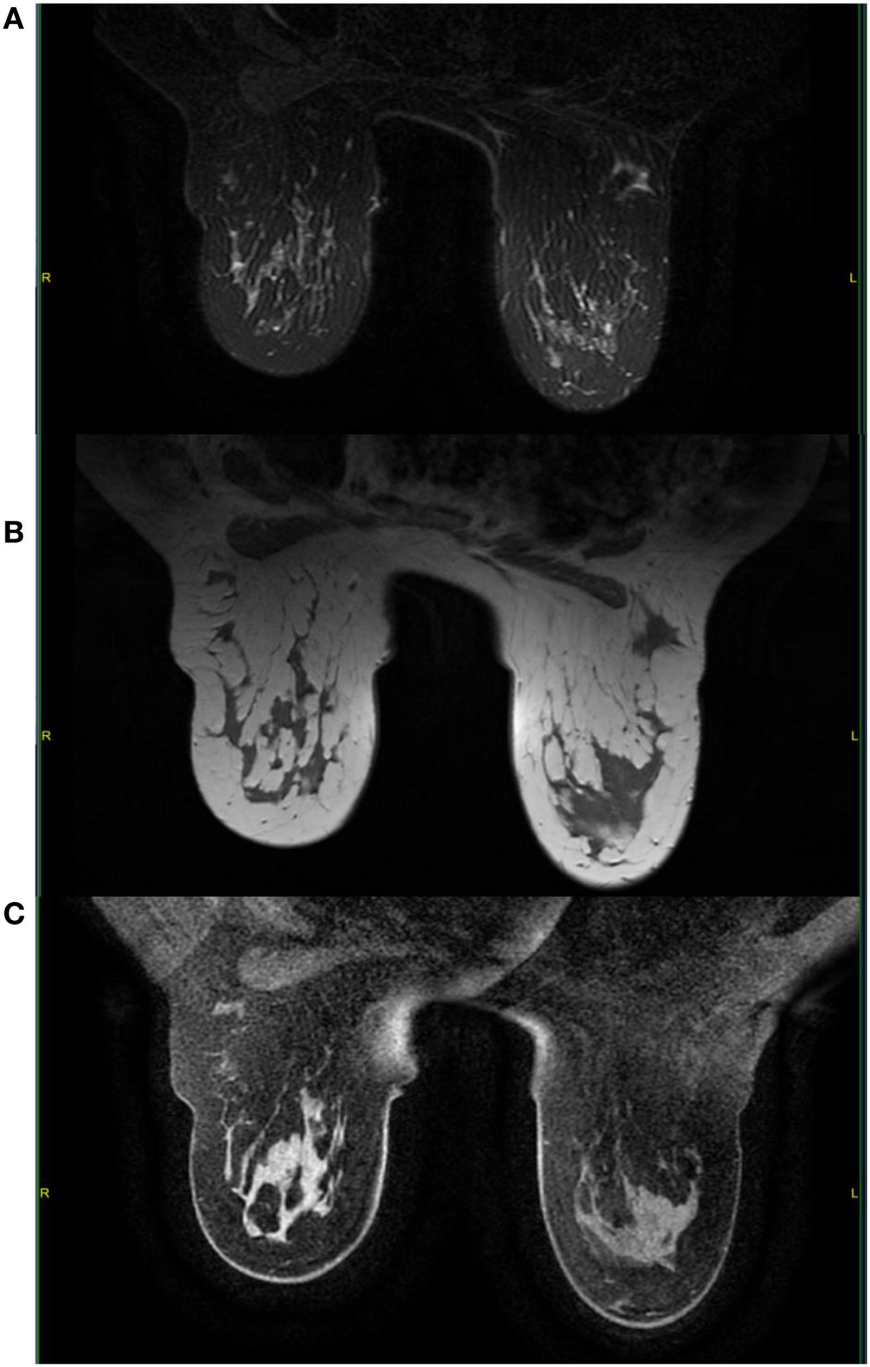
**Here, we observe a complete response of the known palpable lesion in the left breast with absence of contrast enhancement (arrow)**. T2-weighted images with fat saturation **(A)**; T1-weighted images **(B)**; gadolinium-enhanced T1-weighted images with fat saturation **(C)**.

**Figure 8:**
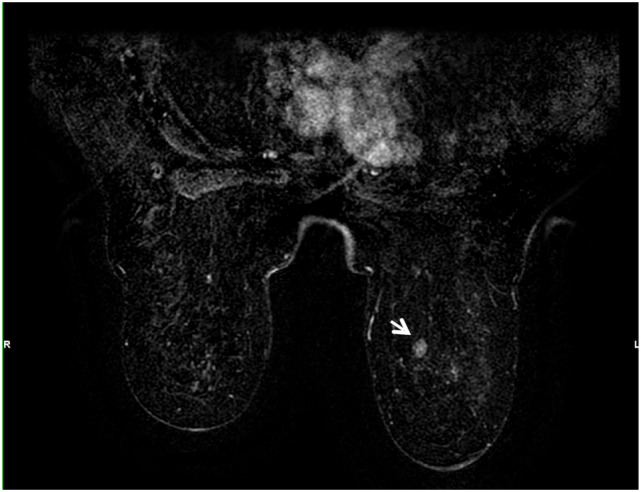
**Gadolinium-enhanced T1-weighted images with fat saturation showing a remaining 1-cm focal enhancement 1 month after therapy (arrow)**.

Salvage therapy with azacytidine was initially administered, but had to be discontinued because of poor clinical condition and development of renal failure. Subsequently, continuous treatment with sorafenib was introduced resulting in a rapid breast ache relief associated with an excellent renal tolerance. Follow-up with breast MRI (Figures 6 and 7) showed partial response of the breast disease within 1 month with complete resolution of almost all known lesions and absence of new ones: only a small fraction of focal contrast enhancements measuring up to 1 cm were observed during follow-up (Figure 8). Although non-specific, we precautiously considered these focal contrast enhancements as malignant since the patient refused a new biopsy.

## Discussion

Granulocytic sarcomas (synonyms: myeloid sarcoma, chloroma) are rare extramedullary tumors composed of malignant myeloid precursor cells and associated mainly with AML although uncommon association with other myeloid malignancies have been described. Rarely, it can arise as a primary tumor in patients with no past or concurrent history of AML. Historically, the term chloroma was coined to describe these tumors because of their greenish color (*chloros* means green in Greek) which is attributed to their MPO content (1–5). GS has a slight predilection toward males (male-to-female ratio, 1:2), and it typically occurs in the later decades of life even though cases have been described in nearly every age group (1–3).

Granulocytic sarcoma affects 2.5–9.1% of patients with AML (1) and can virtually arise in any part of the human body. Most common described sites include the skin, lymph nodes, soft tissues, bones, the CNS, and the urogenital tract (1–6); however, there is some discordancy in the literature when it comes to describing its typical locations. Breast involvement is rare, especially isolated disease. A historical retrospective study in patients with myelogenous leukemia from Hiroshima and Nagasaki (2) depicted breast involvement in approximately 8% of cases. In recent reviews, 153 cases of breast GS have been reported from 1969 to 2005 according to Cunningham (7) and 139 cases between 1980 and 2010 according to Surov et al. (8). In both reviews, most cases were associated with AML, especially as intramammary relapse after therapy, and only a small fraction of these (around 17%) showed isolated breast disease although the majority developed medullary disease somewhere between 1 and 2 years after breast GS diagnosis. Curiously, there is a considerable higher prevalence among females, especially in the pre-menopausal population (90% of cases) (7) in spite of the fact that GS is slightly more prevalent among males suggesting some tropism for the more developed breast tissue which might be independent from classical predisposing factors.

Clinical findings are non-specific and may mimic primary breast cancer. It often manifests as palpable breast nodules that can be either painful or painless and that can involve both breasts. Some may present with skin involvement and/or enlarged axillary lymph nodes. Usually, no nipple retraction or discharge is observed (7–9, 15). As in our case, a thorough investigation of the patient’s medical records is the key for the diagnosis.

Similarly, imaging findings are non-specific, and a low index of suspicion must be kept in mind in patients presenting with breast masses and with a history of hematological neoplasia. Only a few mammogram descriptions exist in the literature (9–11). They usually report non-calcified masses of variable size with hazy and indistinct borders, which can be confused with others malignant breast tumors. Likewise, ultrasound evaluation can be misleading and only a few descriptions have been published so far (9, 10, 12). Generally, lesions are depicted as hypoechoic with microlobulations or spiculated margins. Sometimes it can show prominent vascularity on color Doppler. Unlike mammogram, ultrasound is particularly interesting for younger patients because of the relatively larger fibroglandular-to-fat ratio and the lack of ionizing radiation (12).

On MRI, the lesions normally demonstrate a variable degree of contrast enhancement and are usually hyperintense on T2-weighted images and iso- to hypointense on T1-weighted images (10, 13). In our case, contrast-enhanced images showed a small area of necrosis in the center surrounded by a faint peripheral enhancement in the palpable sore lesion, which is a common finding in other GS locations making it sometimes difficult to distinguish from abscesses (6). Since this particular lesion was painful, there might be some correlation between the presence of necrosis and the clinical features.

Differential diagnosis depends greatly on the age of the patient and on the onset of the medullary disease. Based solely on imaging, younger patients tend to present with benign breast masses such as fibroadenomas and fibrocystic changes. Less common benign lesions include papilloma, hemangioma, and intramammary lymph nodes. More rarely, malignant lesions such as lymphoma and soft tissue sarcomas may be found but those seem to be even rarer than primary breast carcinoma in this age population (12). In older women, the most important differential to bear in mind remains primary breast carcinoma, especially multicentric. Secondarily, breast lymphoma and benign lesions must be considered (6, 10–13).

Final diagnosis is based on histological analysis, which shows tumor cells with evidence of variable degrees of myeloid differentiation. Conventional histological features can be inconstant, therefore misdiagnosis may occur, particularly in poorly differentiated tumors and in patients presenting with isolated disease. GS can mimic a considerable number of other malignancies but in the breast the main misdiagnosis includes non-Hodgkin’s lymphoma (especially large-cell and Burkitt lymphomas), sarcoma, and primary breast carcinoma (especially infiltrating lobular carcinoma) (15). In order to avoid or to reduce this issue, cytogenetic and immunohistochemical techniques as well as flow cytometry are extremely useful (15). These include a vast array of markers which are more or less specific for GS (i.e., MPO, lysozyme, CD68, CD117, etc.), and the combination of two or more helps distinguishing from lymphoma (5, 14, 15). The most common cytogenetic and biochemical abnormalities include *t*(8; 21), inv (16), and cytoplasmic expression of NPM and FLT3-ITD (13). Interestingly, some of these mutations may carry a prognostic value (3, 5, 14).

The mainstay of treatment is systemic chemotherapy with regimens normally used for AML. Several studies and reviews have highlighted the importance of early systemic therapy in order to achieve a long disease-free survival and to avoid AML development or relapse (3–5, 7, 14). There is a lack of clear indication for combination with radiotherapy since survival rates seem to be equivalent, although it might be a useful tool for symptomatic relief or in refractory disease. Moreover, a few retrospective studies have shown a high rate of AML development in patients with isolated GS undergoing radiotherapy or surgery alone. There is no clear consensus if whether or not bone marrow transplantation has an impact even though recent studies have shown a significant survival benefit (4, 5, 14). Finally, promising targeted therapies currently in trial for AML, such as FLT3 inhibitors, farnesyl-transferase inhibitors and histone deacetylase inhibitors might be an effective option against GS (4).

The prognostic value of patients developing GS in the context of AML is somewhat debatable although the historical consensus is that survival is poorer in this population. Other factors must be considered before assessing prognosis, such as cytogenetic profiles and age of onset (4, 5, 14). Conversely, some studies have shown a better prognosis in isolated disease without medullary involvement (4, 14).

## Conclusion

Granulocytic sarcoma of the breast is a rare extramedullary manifestation of AML and other hematologic diseases and sometimes can develop without or prior to medullary involvement. The absence of specific mammographic, sonographic, and MRI features can be very challenging for even the most experienced radiologist. Therefore, a detailed review of the patient’s medical history combined with a low index of suspicion is crucial for guiding toward the proper diagnostic procedures. These include histological examinations with ancillary techniques that are paramount to confirm the diagnosis and to assist in planning the subsequent treatment.

## Ethics Statement

Written informed consent was obtained from the participant of this case report.

## Author Contributions

VF and QV are the main authors; JJ is the corresponding author, and he also provided corrections and acted as one of the main consultants/experts; FK acted as consultant/expert; AF acted as consultant/expert; and HH provided corrections and acted as one of the main consultants/experts.

## Conflict of Interest Statement

The authors declare that the research was conducted in the absence of any commercial or financial relationships that could be construed as a potential conflict of interest.
